# American Academy of Dental Science

**Published:** 1895-12

**Authors:** 

**Affiliations:** American Academy of Dental Science


### AMERICAN ACADEMY OF DENTAL SCIENCE.

THE regular meeting of the American Academy of Dental Science was held at Young's hotel, Boston, April 3, 1895, at six o'clock.

The paper for the evening was read by Dr. Charles A. Brackett, of Newport, R. I.; subject, "Assistants and Assistance."

(For Dr. Brackett's paper, see page 736.)

### DISCUSSION.

*Dr. Brackett.*—Certainly all the ways of transacting the business of the world are undergoing modification. Not infrequently, when stopping in the city at the Thorndike, I have been assigned to a room which overlooked the doors that open into Park Square from the Providence Depot, and in observing the people coming in to their day's work on the early trains I have been very much impressed by noting what a large proportion of them are women, the majority of them apparently full of health and cheerfulness. It is impossible to go about in any of the business districts of this great city without noticing the fact that wherever there are books to be kept or letters written, women are doing a very considerable share of the work. I am a thorough believer in the dignified right of women to do all these things and many others. I am a thorough believer in the perfect propriety of their being graduates and independent practitioners, but there are many women of ability who perhaps would prefer not to assume the conduct of an independent enterprise, who may find in the assistant's place great opportunity to be useful to themselves and to others. I think there are some sound principles of social economy involved in this. I am sorry to say that I am only a very superficial student in political economy, or even in general economy. I must confess that I know very little about them, but it seems to me genuine economy in a man, when he has attained his skill in doing certain technical work, to employ labor to do that routine work in which less skill is required, or labor requiring another kind of skill. I have among my patients a man who has vast business interests, not of his own, but he has millions of property in his hands for his direction, and this property is particularly liable to injury from the elements: it is at risk at sea. He is a domestic man, and his wife sometimes asks him to do little things about the house. He does whatever he is asked, but occasionally, when he is fixing up new curtains or something of that sort, he will remind his wife that she is employing rather an expensive man for that kind of work. That is the idea that in a sense applies to our work, and it seems to me the more we can cultivate this system, and not have it get beyond the demands of meeting the volume of business at hand, the more everybody is advantaged. There is as sound a principle for gain for the employer on a small scale as there is for the Fall River manufacturers who employ hundreds or perhaps thousands of operatives, or for the New York and New Haven Railroad Corporation in its great system, so that for the principal and employé it is a decidedly beneficent thing. Then when it comes to the service which is rendered to those who are the recipients of the service, they are greatly favored; they are acceptably favored. The day has gone by, I think, in most busy offices when the patient expects to see about the office no one but the single practitioner himself, and we should be surprised nowadays to have a patient say to us, "Now, this denture that I am to have you will do all with your own hands, won't you?" We do not hear that now as we did years ago. The patient recognizes the principle involved, that the directing mind need not necessarily have its own hands accomplish the work. I think the principles that I have tried to state, however poorly, are sound principles. I have endeavored to put this matter before you in a suggestive way. I speak not only to those of you who employ assistants, but I wish to put these matters before those of you who are struggling to meet the demands of a busy practice, who have arrived at that stage where the most unwelcome communication you receive is an application for an appointment, and the most dreaded sound is the ring of the door-bell, and to say to you that by the employment of assistance you may get rid legitimately and advantageously to all concerned of a share of the routine burden of work.

*Dr. Eddy.*—As you all know, I thoroughly believe in assistants, both lady assistants and professional graduates. There are many ways in which they can be of help to you. To begin with, there is a noticeable improvement in the neatness of the office, as every office shows at once the touch of a woman's hand in the case and cleanliness in the arrangement of things about the room, and this neatness reacts upon the operator in the matter of dress. There is also a certain amount of refinement of manner in the woman, and that cannot fail to reach the operator and promote carefulness in his personal conduct and also in the expressions he uses before his patients. Again, the watchfulness and helpfulness of a lady at the chair begets self-confidence in herself, which, in turn, is fostered in your patient, who develops confidence in the assistance, and they soon become perfectly willing to place themselves in the assistant's hands for whatever part of the work is intrusted to the assistant. I have my assistant meet all patients and attend to all formalities of receiving them and getting them ready for the chair; also, make appointments; also, when a patient comes for the first time, and the assistant takes a chart and makes a thorough examination of the patient's teeth, charts all cavities, and notes the doubtful ones, there is a great deal of time saved in knowing that you have seven cavities to deal with instead of seventeen. My oldest lady assistant treats all my dead teeth. After I remove the pulp she follows it up with whatever treatment I may suggest, fills the canals with gutta-percha, and the case comes back into my hands at a subsequent appointment with the main cavity ready to be filled.

I have found ladies more helpful as assistants than graduate dentists. After a graduate has been with you for a while his time is more and more engrossed with the patients which his own tact and ability have enabled him to acquire, as well as those you have placed into his hands. You do not feel like asking him to do those things that belong properly to a helper, and by the end of the first year they are of no assistance whatever. The lady does exactly what you tell her to do, and is more faithful in all her service; does not introduce a method of treatment you know is obsolete and from experience not satisfactory. One way in which they can save you considerable time is in making alloy or cement fillings. We have electric bells at all the chairs, and a hotel annunciator on the wall to tell which the call is from, there being five chairs, four operators, and one girl attends to two chairs. If we want filling material, a girl is called and told what is wanted, and by the time you are ready for it she has it all prepared. In preparing for crown-work there are cases where you would like to have some one mix your cement for you, and a lady assistant, with a little practice, can do this very satisfactorily.

What does all this help and avoidance of interruption mean? It means time saved that is valuable and that can be given to your patients. As the years go on, a man's reputation and practice should increase, and the only way he can obtain the requisite relief for exercise, social and mental stimulus, is by assistants. Again, it is a great precaution to have a lady assistant. In giving anæsthetics to a lady a man with a small practice, in a city like Providence, is sometimes placed in an embarrassing position, and there are times when a lady assistant could be of great service to the operator.

As to the graduate assistant, a man at graduation is full of text-books, but not generally posted in professional readings. Seven out of eight of them are wholly without office experience. He enters an office of a busy practitioner. He exchanges text-books for years of experience. The old and new are brought together, and are both benefited. No man is wholly self-made. There are not all the elements in a man to wholly develop him; he has undeveloped faculties, or faculties apathetic. Co-operation develops faculties and activity, and a longing for the truth. The old man is pulled from the rut; the young man moves steadily, because he has a balance-wheel and a regulator to carry him over the dead centre, and take up and smooth out his erratic motion. Men do business alone and succeed, but the highest success comes by combination; evidence your great department stores and combination of bankers, etc.

Professional men to-day need more correct business methods. Different men in an office look at the same subject from different points; it is the combination of the photographs of the same subject that makes the true picture.

The advantage of assistants from a pecuniary point of view Dr. Brackett has already suggested to us.

All these matters pertaining to the assistants and how best to make use of them bring added cares and responsibilities to the dentist, but who would not prefer in this busy, rushing nineteenth century to go out like the bursting of an incandescent lamp than to drown in his own oil like a tallow dip. We have only one life to live, and we all want to do our best with it. I don't see how it is possible for a man in a busy practice to do justice to himself and his patients without making use of assistants.

*Dr. Clapp.*—Dr. Brackett has told his story so gracefully that it is rather embarrassing to attempt to add anything to it. I will fully endorse every word that he has spoken, and just give one more duty that I place upon my lady assistant. This lady has been with me for twelve or thirteen years, and one of the things from which I receive very great comfort is in her attention to children and nervous patients. For instance, if I am at work on a child, she will read or tell the child stories that are suitable to its age, and these are the best anæsthetic, the best obtundent with which to handle children, as far as my experience goes. I thoroughly believe in it. Time and time again I have a child in my chair, and the lady devotes her attention to managing the child while I do the work and keep my mouth shut. Oftentimes for ladies, young ladies, ladies of middle age, or elderly ladies, she reads chapters from books or short stories, and a two-hour appointment passes almost before they know it. I would recommend to all of you who have lady assistants to try this method, for I am sure you will find that you will get very great comfort. I would like to emphasize one point Dr. Brackett has touched upon, and that is this: how anybody with a practice that takes his whole time and a little more can afford to be without an assistant at the chair is something that very much puzzles me. One can do from one quarter to one-half more work with the assistance of a lady than he can do alone. For instance, your patient is in the chair; every accessory to the operation is attended to by the assistant; you adjust the rubber dam, you prepare your cavity. Before it is quite completed you have determined with what you will fill that cavity. If it is amalgam, you tell your assistant that you want a medium, small, or large filling prepared of such an alloy. It is prepared and placed on the table before you, and when you lay down your excavator, after having made the last cut, without a second's delay you proceed to insert the filling. The same thing, of course, can be done with gutta-percha or cement as with the amalgam filling. The only objection to this method is that you wear your life out a little quicker; you won't live quite so long as if you stopped and took a little rest, instead of having things arranged so as to keep you working every minute of the day.

*Dr. Andrews.*—In a general way, Dr. Brackett's description of the lady in his office, and her duties, corresponds so nearly to the lady in my own office that I will simply say he has told you my own experience better than I could myself. My opinion coincides with that of Dr. Clapp and Dr. Brackett, that no busy dentist can afford to do without a lady assistant. I was very fortunate, perhaps, in securing one of the best,—I don't think it would be possible to have a better one. I would like to call on Dr. Cutter, who has had some experience with lady assistants.

*Dr. Cutter.*—I can add but little to what has already been said with reference to the helpfulness of lady assistants. The one that I have is invaluable to me. She makes all my regulating appliances and also relieves me of much care in adjusting them for my patients. I do not see how I could get along without her.

*Dr. Ames.*—You are all telling about the prizes which you have. I want to tell about mine. I have two lady assistants. I had a man in my laboratory for several years that I considered a very fine plate-worker. He was a young colored man who had worked at it for a long time, and a couple of years ago he died very suddenly, and for a long time I could not find any one to fill his place. I finally decided that I would have to teach some one, so I took a young girl who was a niece of my cook. She was a young Irish girl, with a fair education, and was a nice, respectable young woman about twenty years old, and I commenced to teach her to make artificial teeth. She took hold readily and learned very quickly, and now she does all the work that is done in my laboratory to my satisfaction. My other assistant is very valuable to me, shows a great deal of tact in receiving patients, takes care of the office nicely, looks out for my instruments a great deal better than I could myself, and I think she is the best lady assistant I have ever seen. I never had a graduate assistant.

*Dr. Eames.*—It is difficult to add anything to such a complete paper on the duties of assistants, but, in a general way, I think, whoever has had such service, and attempts to get along without it, soon finds how much help the assistant was to him. It is almost impossible, after being accustomed to such assistance, to get along without it. I am indebted to Dr. Clapp for suggesting what he has told here to-night in the way of reading stories to children and to older people, and I have been following the suggestion for the last five years, with the greatest satisfaction. I have been fortunate in having my own sister as assistant, and I intrust to her the keeping of my books, the making out of my bills, and almost my entire correspondence. In the matter of filling the root-canals, I have not gone so far as to trust that to any one. It seems to me that it is an operation needing special skill and a knowledge of just what is best to be done. I would almost say that I would trust the filling of a cavity to an assistant sooner than the dressing of a canal, but I can see, very readily, how one could save a great deal of time if the assistant were competent to do it. In regard to the diagram and examinations of mouths, I have special hours in which I see patients for examination. I have small diagram-cards on which the result of the examination is noted, and at the time of the examination I try to estimate the time it will take to complete the work, and all remarks which I consider necessary to the case are put on this slip of paper and kept by the operating-case for reference until the work for the patient is completed. This, with the record of each visit and amount charged, is transferred to a larger diagram, on which fuller remarks are entered and all are subsequently posted into a book. In the matter of introducing gold to a cavity, I am sure that I lessen the time of operating one-half. The annealing of gold and placing it in the cavity is done by the assistant. I simply nod my head when I wish it to be placed in the cavity and with the plugger point to the exact part where it is to be placed. The last touch of the instrument is understood by the assistant to be the spot where it is to be introduced. I would say that I find it of the greatest value to have duplicate instruments, and when I am through with certain excavators the assistant takes them away, and duplicate instruments, napkins, etc., are furnished, and the chair is in readiness almost instantly for the next patient.

*Dr. Ainsworth.*—I don't know that I can say anything in addition to what has been said. I have for a number of years made use of an assistant; not in all the ways that have been referred to to-night, but I can readily understand that the right person could render the assistance satisfactory in most of the cases spoken of. I should not know how to get along without an assistant, now that I have come to rely so much upon one.

*Dr. Werner.*—I think that must be the universal report of all those who have had assistants. From the first I have had my lady assistant assist at the chair, passing gold to the cavities. I talked not long ago with a dentist who has had an assistant for a long time, and he asked, "Did you say your assistant helps you at the chair?" I replied, "Why certainly, what would I have her for if not for assisting me at the chair?" He then wanted to know if some of the patients did not object to this, and I could record but one instance in which a patient objected, and that was mainly from a misapprehension of the duties of the assistant, and not being accustomed to having any one around besides the dentist. At the second sitting I asked the patient if she had any objections to the assistant helping me during the operation, and she replied, "Oh, no, I have got used to it now."

The first instruction I would give a new assistant would be in picking up things with the foil-carrier. I should at once set her to practising picking up small pieces of paper with the foil-carrier, and show her how to do it in such a way as not to obstruct her view or mine. That seems to be a simple thing for them to learn, and yet it is of great assistance to the operator. I find that they are very faithful and accurate in book-keeping. To my surprise the lady assistant I have now, after an experience of only three months, is making out and receipting all bills, and for months made but one trifling mistake.

*Dr. Clapp.*—I want to add just one word in connection with this subject of assistants, and that is, the advisability, I might say necessity, of having large operating-rooms. I do not believe in having an operating-room that is not more than eight feet square, and confining three people in that room for the greater part of the day. In the interest of health it seems to me that an operating-room should not be less than fifteen feet square.

*Dr. Werner.*—You cannot always have that in an expensive locality of a city. There should be an electric fan in every dental office. They are a comfort, ventilating and purifying the atmosphere, preventing odors of anæsthetics or medicaments about the room or clothes of the operator; they are noiseless and inexpensive.

*President Smith.*—If no one else wishes to speak on this subject, I will ask Dr. Brackett if he cares to say anything in closing the discussion.

*Dr. Brackett.*—I will detain you but a few minutes. I wish to express this thought: The modern busy dentist, whatever his ambition and ability, and with all the help that he can advantageously employ, is still able to care for only a limited amount of dentistry. From this point of view one readily sees the necessity for economy in the expenditure of our time and energy.

I neglected to mention the practice of reading in the office, referred to by Dr. Clapp, and which I have followed somewhat for years with a great deal of comfort.

William H. Potter, D.M.D.,
*Editor American Academy of Dental Science.*

